# Integration of visual and whisker signals in rat superior colliculus

**DOI:** 10.1038/s41598-018-34661-8

**Published:** 2018-11-06

**Authors:** Saba Gharaei, Ehsan Arabzadeh, Samuel G. Solomon

**Affiliations:** 1 Discipline of Physiology, School of Medical Sciences, The University of Sydney, Sydney, Australia; 2 Eccles Institute of Neuroscience, John Curtin School of Medical Research, The Australian National University, Canberra, Australia; 3 Australian Research Council Centre of Excellence for Integrative Brain Function, The Australian National University Node, Canberra, Australia; 4 Institute of Behavioural Neuroscience, University College London, London, UK

## Abstract

Multisensory integration is a process by which signals from different sensory modalities are combined to facilitate detection and localization of external events. One substrate for multisensory integration is the midbrain superior colliculus (SC) which plays an important role in orienting behavior. In rodent SC, visual and somatosensory (whisker) representations are in approximate registration, but whether and how these signals interact is unclear. We measured spiking activity in SC of anesthetized hooded rats, during presentation of visual- and whisker stimuli that were tested simultaneously or in isolation. Visual responses were found in all layers, but were primarily located in superficial layers. Whisker responsive sites were primarily found in intermediate layers. In single- and multi-unit recording sites, spiking activity was usually only sensitive to one modality, when stimuli were presented in isolation. By contrast, we observed robust and primarily suppressive interactions when stimuli were presented simultaneously to both modalities. We conclude that while visual and whisker representations in SC of rat are partially overlapping, there is limited excitatory convergence onto individual sites. Multimodal integration may instead rely on suppressive interactions between modalities.

## Introduction

Environmentally significant events are often simultaneously detected by more than one sensory organ. The capacity to integrate information across the senses improves detection, localization and identification of these events^1–3^. While a number of brain areas are likely to be involved in multisensory integration^4–6^, the most studied is the superior colliculus (SC), an evolutionary ancient midbrain structure that receives inputs from multiple sensory modalities and helps orient subsequent behavior^7–9^.

The functional properties of multisensory integration in SC has primarily been explored in the context of audio-visual integration in cat, and to a lesser extent in primate. Many SC neurons show excitatory response to both auditory and visual stimuli. These multisensory neurons usually show supralinear response to combined visual and auditory stimuli^1,10–13^. Among neurons that show excitatory responses to only a single modality, multisensory presentation can nevertheless modulate responses to this modality, usually by suppressing their response^14–17^.

As with cat and primate, rodents are also capable of combining information across senses, and show performance benefits to multimodal stimuli^6,18–23^. In guinea pig SC, dark rearing leads to degradation of spatial tuning and topography of multi-unit auditory responses, implying that map elaboration is dependent on coincident visual and auditory information about a stimulus^24,25^. In rat SC, as in cat and primate, audio-visual interactions are widespread, at least in younger adult animals, where they are usually additive or supra-additive^26^. As in cat and primate, some of these neurons appear unisensory when stimuli are presented separately to each modality, and multisensory interactions may only become apparent during multisensory presentations^26^. Yet while work on multisensory integration in cat and primate has focused on SC, multisensory integration in rodents has mainly been studied in cortex, in both higher-order cortical areas^6,27–30^ and in primary cortical areas traditionally considered unisensory^31–33^.

In rodents, as in other mammals, the extensive visual inputs to SC that arise from both retina and visual cortex^34–36^ are complemented by auditory, and somatosensory (particularly whisker) inputs^37–39^. The topographic representations of visual and whisker inputs are in approximate spatial register^40,41^ and this suggests that the SC of rodents may be a site for visual-whisker integration, but whether and how these representations interact is not clear. Here we first establish the spatial and temporal overlap of visual and whisker inputs to SC, and show that individual sites rarely exhibit excitatory responses to both modalities. We then develop a generalized model framework to characterize the interaction between the two modalities, and show robust cross-modal suppression.

## Methods

### Ethical approval

Adult male hooded rats (Long Evans, n = 11, weighing between 243 and 447 g, age 6–17 weeks) were used in the current study. Procedures were approved by institutional (University of Sydney) Animal Ethics Committee, and conform to the Society for Neuroscience policy on the use of animals in neuroscience research.

### General

Each animal was initially sedated with an intramuscular (I.M.) injection of a combination of ketamine (80 mg/kg) and xylazil (6 mg/kg). We gave preoperative intramuscular injections of dexamethasone (0.3 mg/kg; Maine Pharmaceuticals, VIC, AUS) to reduce inflammation. The trachea was then exposed and an endotracheal tube was inserted to allow artificial ventilation, and the head was placed in a stereotaxic frame. Post-surgical anesthesia was maintained by isoflurane (0.5–1% in a mixture of 1:1 nitrous oxide and oxygen). The electrocardiogram (ECG) and SpO2 were monitored continuously. The animal was artificially ventilated, with a 60:40 mix of N_2_O and Carbogen, so as to keep end-tidal CO_2_ near 33 mmHg. ECG signals were monitored to ensure adequate depth of anesthesia. Body temperature was kept near 37 °C with the use of a heating blanket. At the end of the experiment the animal was euthanized with an intraperitoneal injection of 500 mg/kg sodium pentobarbitone (Lethobarb; Verbac Australia, NSW, AUS). To confirm the electrode position in SC, the animal was perfused transcardially with 0.9% sodium chloride solution and then 4% paraformaldehye in 0.1 M phosphate buffer. Coronal sections (50 μm thick) were cut on a freezing microtome and were stained with cresyl violet to reveal Nissl substance.

### Electrophysiology

**Figure 1:**
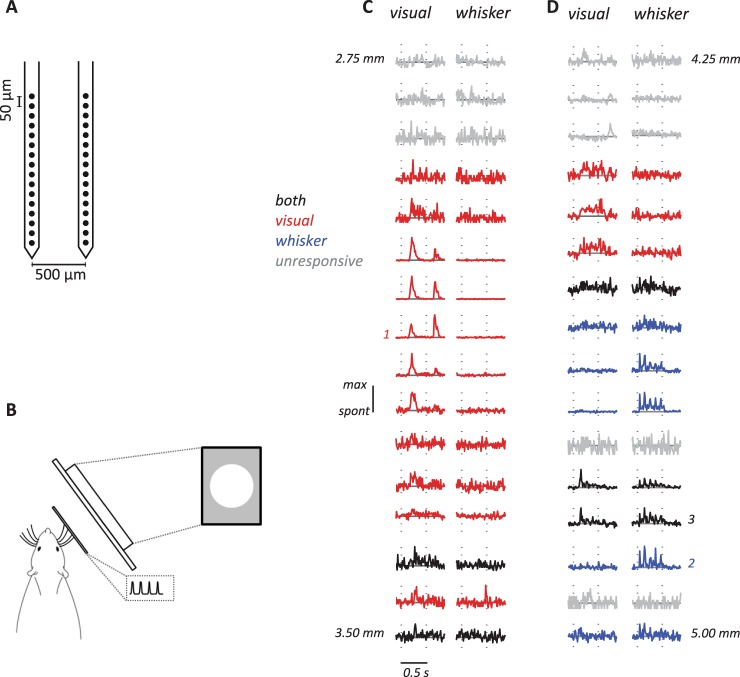
(**A**) Schematic representation of the dual-shank electrode array. (**B**) Schematic representation of the experimental set-up. (**C**,**D**) Example peristimulus time histograms (PSTH; bin width 10 ms) to visual or whisker stimulation recorded at different depths. Each of C and D shows a simultaneously recorded set of responses. Visually responsive electrodes are drawn in red and are primarily located in the superficial and intermediate layers. Whisker responsive electrodes (blue) are in intermediate layers. Response to both visual and whisker stimulation (black) are also observed across layers of SC. (**C**) Responses on an electrode shank placed such that the most superficial site was a depth of 2.75 mm (the distance between each site is 0.05 mm). Only one of the two 16-channel shanks is shown. The dashed vertical grey lines indicate the onset and offset of the stimulus (stimulus duration = 500 ms). The response of each site has been normalized to the maximum of PSTH for that site. (**D**) Responses on a shank placed deeper into SC (4.25 mm).

A craniotomy of ca. 3 mm was made, centered 6.8 mm posterior to Bregma and 1.5 mm lateral to the midline. In 7 animals, extracellular recordings were obtained using high-impedance single electrodes (3–5 MOhm, Thomas Recordings). The analogue signals from the electrodes were amplified, band-pass filtered (0.3–10 kHz) and sampled at 48 kHz by the same computer that generated sensory stimuli. In a vertical penetration of the electrode, the surface of SC was localized as previously described in rats^42^ and based on observing the first visually responsive neuron. Spike waveforms likely to arise from a single unit were identified using on-line principal component analysis, and refined off-line. The timing of waveforms was recorded with an accuracy of 0.1 ms. In 4 additional animals, a 2 × 16 dual-shank linear silicon probe (Neuronexus; A2x16-10mm-50-500-177-A32), with an inter-contact distance of 50 μm and inter-shank distance of 500 μm, was inserted perpendicular to the cortical surface (Fig. 1A). Signals from each contact point were amplified, bandpass-filtered (0.3–5 kHz), and digitized at a rate of 24 kHz by an RZ2 real-time processor (Tucker-Davis Technologies, FL, USA). The function *findpeaks* in the Matlab environment (MathWorks, Natick, MA, USA) was used to identify candidate waveforms with peak amplitude that exceeded 4 standard deviations (SDs) of the raw signal on the relevant channel. We recorded multi-unit activity from a total of 672 electrode contacts at 21 recording sites. We also recorded 108 single-units using the single electrodes, 65 of which were responsive to at least one of the stimuli we used and were therefore included in analyses. All analyses were performed in the Matlab (Natick, MA) environment.

### Stimulus presentation

Visual stimuli were generated by a G5 Power Macintosh computer using custom software (EXPO; P. Lennie, Brain & Cognitive Sciences, University of Rochester, USA); they were drawn with eight-bit resolution using commands to OpenGL. Visual stimuli were displayed on a calibrated LCD monitor (ViewSonic 703b; width 39 cm, height 40 cm; refresh rate 85 Hz, mean luminance ~45 cd/m^2^) viewed directly at a distance of 30 cm in a dimly lit room, and centered on the grand mean of multiunit activity from recording electrodes after broadly mapping the receptive fields of multiunit activity at each responsive site. The visual stimulus was a circle with increment or decrement in light from the mean luminance (up to 100° in diameter: median = 87°; mean = 75°). Somatosensory stimuli were waveform trains generated by the same program and communicated through the sound card. These waveforms were used as inputs to the controller of a mini-shaker (Brüel & Kjær; vibration exciter 4810, amplifier 2718) attached to a vibrating metal mesh (2.5 cm × 2 cm). The mesh, placed at a distance of 0.5 cm from the base of the shaker, contacted whiskers of the contralateral whisker pad and moved along the vertical axis. Most whiskers contacted the mesh within 0.3 cm of the base of the whisker pad. The visual and somatosensory stimuli were close by in space (spatially aligned). Given the multisensory nature of SC, the following two control tests were performed to ensure that the responses to whisker stimulus were not elicited by the auditory noise from the vibrating mesh or its movement through the visual space: i) for the sites that responded to the whisker stimulus, the experiment was repeated with the mesh placed close to the whiskers without contacting the whiskers; ii) at the end of a subset of experiments, the whiskers were trimmed and the mesh was placed at the same location as before. In both of these control tests, spiking activity in response to the vibrating mesh disappeared indicating the tactile nature of the whisker responses. Neurons in intermediate layers of SC prefer multi-whisker vibrations^43–45^, so we used large visual flashes and multi-whisker vibrations^6,46^ (Fig. 1B). Each set of stimuli, which always included baseline measurements (blank screen of the mean luminance, no whisker vibration), were presented in pseudo-random order.

All recording sessions included a stimulus set where visual and whisker stimuli were presented in isolation for 0.5 s, at maximum intensity (visual Michelson contrast ~1; whisker vibration amplitude = 2.43 mm) with inter-stimulus interval of 0.5 s. Between trials the monitor was held at the mean luminance and the mesh was still. Responses were obtained for a median 100 repetitions of each stimulus (mean 93, SD 17.9, range 34–100). We presented each neuron with a white and a black stimulus and chose the one with higher onset responses for the multisensory condition. In a subset of the above sessions, the 25 combinations of 5 visual contrasts (0, 0.12, 0.25, 0.5, 1.0) and 5 whisker vibration amplitudes (0, 0.30, 0. 60, 1.35, 2.43 mm) were interleaved in pseudorandom order (inter-stimulus interval 0.3 s). Responses were obtained for 50 repetitions of each stimulus. The visual stimulus lasted 0.2 s and the whisker stimulus 0.12 s. The onset of visual stimulus was 0.08 s before onset of whisker stimulus. This onset asynchrony was chosen based on the neuronal response latencies measured in a pilot experiment, as previous work showed that appropriately shifting stimuli in time optimizes the opportunity for multisensory interactions^47–50^. In separate measurements, we presented the visual and whisker stimuli synchronously in the multisensory condition.

### Analysis

Peristimulus time histograms (PSTH, bin-width 10 ms) were generated for responses to each stimulus. Spike counts for each trial of each stimulus were calculated over the duration of the stimulus presentation. Many sites showed ‘off’ responses (increase in activity at the offset of the stimulus; cf. Wang *et al*.^51^); we have not considered them because the source of the ‘off’ response is not clear (for example, it may arise in rebound activity after hyperpolarisation, or convergence of parallel afferent pathways). Unless otherwise stated, we characterized the evoked response (the difference between activity during the stimulus and the spontaneous activity, which was estimated from the period when the monitor was held at the mean luminance and the mesh was still). All analyses except those used to classify neuronal responses and model fitting (described below) therefore rely on the difference between mean spike count over the duration of stimulus presentation, and activity during interleaved ‘baseline’ measurements. Response latency was defined as the first occurrence, after stimulus onset, of two consecutive bins (here bin-width of 5 ms) with significant responses (Student’s t-test; *p* < *0.05*).

### Classification of neuronal responses

To characterize neuronal responses to visual and whisker stimuli, we used a nonparametric, receiver operating characteristic (ROC) analysis^52^. The ROC provides a method for deciding whether spiking activity is responsive to sensory stimulation. Formally, the ROC estimates whether an ideal observer could classify whether a given spike count was recorded in one of two possible conditions (here stimulus present, or absent). In each case we compared the vectors of trial-by-trial spike counts obtained in the 500 ms before and after stimulus onset (i.e. the duration of the stimulus). The overlap between the two spike count distributions was quantified by applying criterion levels in steps of 1, ranging from the minimum to the maximum observed spike count. Each criterion yields a hit rate (stimulus present, appropriately detected) and false-alarm rate (stimulus absent, inappropriately detected). Plotting hit-rate against false-alarm rate for every criterion yields the ROC curve. Trapezoidal integration of area under the ROC curve provides a single number that falls within the range of 0 to 1. A value of 0.5 indicates no difference between the two distributions of spike counts (and thus no measureable sensory response). A value of 1 indicates no overlap between the two distributions (and thus perfect detection by the ideal observer). The statistical significance of the ROC value was determined by bootstrap analysis, such that a synthetic ROC was calculated for each of 1000 pairs of distributions in which the labels (stimulus present or absent) were randomly assigned to each observed spike count. The fraction of synthetic trials with ROC values greater than the observed value provides the significance value of that ROC.

### Quantifying multisensory integration

To quantify whether the responses to the multisensory condition was linear, sublinear or supralinear, we first compared multisensory response with that predicted by summation of responses to visual- and whisker stimuli presented alone.

For comparison with previous work^7,14,47,53^, we calculated an “interactive index” (ii):

1 $$ii=\frac{CM-S{M}_{max}}{S{M}_{max}}\times 100$$

where *CM* is the mean response evoked by the multisensory stimulus (visual plus whisker), and *SM*_*max*_ is the mean response evoked by the preferred single modality stimulus (visual or whisker). The interaction index characterizes how the multisensory response differs from the largest evoked unisensory response. A positive *ii* value indicates increased response in the multisensory condition, whereas a negative *ii* indicates a reduction of response in the multisensory condition. Statistical comparisons between these conditions were performed using a nonparametric Wilcoxon rank sum test, as the data was not normally distributed, according to the Kolmogorov-Smirnov normality test.

### A simple model of multisensory integration

The intensity response function of sensory neurons can often be characterized by a sigmoidal function. Parameters of the sigmoidal function, such as baseline, inflection point, response range and saturation quantify key aspects of the neuron’s response profile. These features are also quantifiable by classes of normalization models (for example Carandini & Heeger^54^). These normalization models provide a computational framework for multisensory integration that can account for key principles in multisensory integration, including the principle of inverse effectiveness, and the spatial principle^55^. Normalization models also provide a compact description of how neurons weigh inputs from each modality, across a range of stimulus strengths. To characterize neuronal responses to a range of stimulation amplitudes across two modalities, we used the following normalization equation, based on Naka-Rushton function^56^:

2 $$R(V,S)={R}_{{\rm{\max }}}\frac{{({w}_{1}V+(1-{w}_{1})S)}^{n}}{{({w}_{2}V+(1-{w}_{2})S)}^{n}+{{M}_{50}}^{n}}+b$$

where *V* is the strength of the visual input, and *S* is the strength of the somatosensory input, each normalized to the range 0 to 1; The numerator is a weighted linear sum of these sensory inputs, with the parameter *w*_1_ controlling the relative excitatory drive assigned to each modality. The denominator contains two terms: one is again a weighted sum of visual and whisker inputs, their relative strength controlled by the parameter *w*_2_; the second term, *M*_50_, is a constant that defines the weighted input strength at which half maximal response is attained. Additional parameters provide an expansive nonlinearity (*n*), scale the response (*R*_*max*_) and provide a maintained discharge rate (*b*). In total there are 6 free parameters; for each site we found the combination of parameters that best predicted response by minimizing the square error between the model predictions and observed responses, using the nonlinear function “fit” in the Matlab environment. There are separate weights in the numerator and denominator because the normalization pool may weigh contributions differently.

For each site we found, the best predictions of the model described above, as well as a reduced model that did not contain the normalization term (the denominator). The reduced model has 4 free parameters, lacking *w*_2_ and *M*_50_. To establish the improvement in model predictions gained by adding the normalization terms (and thus adding two extra free parameters), we used a modified version of the Akaike Information Criterion (AIC)^57–60^. AIC is refined to correct for small data samples; If ratio of n/K < 40, then AICc is calculated using the following bias adjustment:

3 $$AIC=n\times \,\mathrm{ln}\,\frac{RSS}{n}+2k,AICc=AIC+\,\frac{2k(k+1)}{n-k-1}\,$$

where K is the number of free parameters, n is the number of data samples and RSS is the residual sums of squares. The best model is the model with the lowest AICc (or AIC) score. Note that the AIC and AICc scores are ordinal and do not convey any information on their own. They are merely a way of ranking different models.

## Results

We recorded spiking activity in SC of Long-Evans rats, and measured responses to visual stimuli and whisker vibrations. In the following, we first characterize responses to visual and whisker stimuli presented in isolation, and then ask how these responses are changed during simultaneous presentation. We made systematic measurements of spiking activity across layers of SC, using multichannel linear probes. To characterize response at multiple recording sites simultaneously, we used large stimuli that contacted most of the contralateral whisker pad, and extended across much of the contralateral visual field.

### Depth distribution of visual and whisker activity

Figure 1C shows measurements of (multi-unit) spiking activity across 16 channels of an electrode implanted into superficial SC. Activity at the 3 most superficial sites is not modulated by either whisker or visual stimulus, and these sites are likely to be located above SC; deeper sites show primarily visual responses (usually responding to both onset and offset phases of the bright flash shown here). Figure 1D shows responses obtained at a deeper penetration into SC. In this case, activity at each contact point on the array can be sensitive to visual stimuli, whisker stimuli, or both.

**Figure 2:**
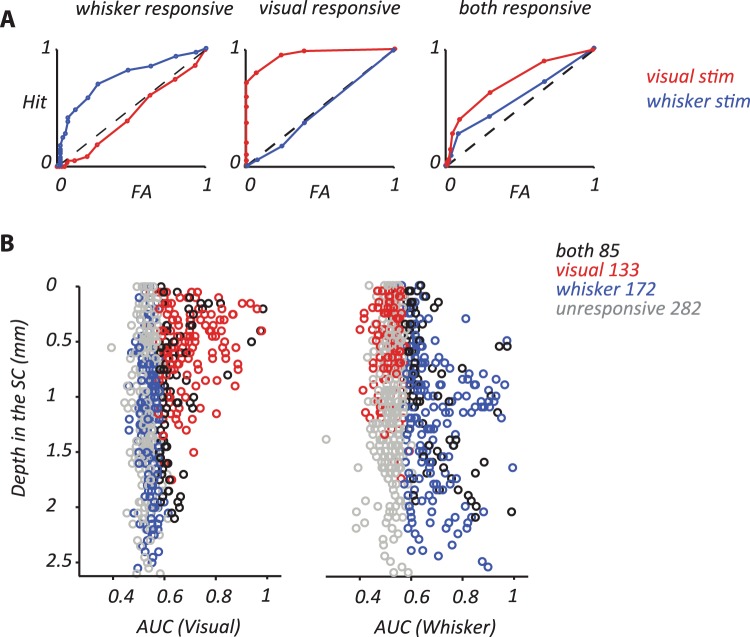
Response characterization of SC spiking activity using ROC analysis. (**A**) Receiver operator curves (ROC) analyses for a whisker-responsive recording site, a visual-responsive site and a site that responded well to both whisker- and visual stimuli. ROC analyses quantify the overlap in responses during stimulus presentation and maintained activity in absence of stimulus. Each point indicates the hit and false-alarm rates for one response criterion (see Methods). The Area Under ROC (AUC), calculated from these curves, characterizes the capacity of neuronal response to signal the presence of the relevant stimulus, and has values between 0 and 1. AUC of 0.5 indicates complete overlap between response distributions and no stimulus sensitivity. AUC of 1 indicates complete separation of response distributions. (**B**) Dependence of AUC on depth of recording site within SC, for visual (left) or whisker (right) stimulus presentation. Sites with significant response to both visual and whisker stimulation (black circles) are replicated in both plots. Visually responsive electrodes with high response rate (higher ROC values) were mainly in superficial layers. Whisker responsive electrodes with high response rate were mainly in intermediate and lower layers.

To define activity as primarily visually driven, primarily whisker driven, neither or both, we used an ROC analysis of responses obtained across multiple trials (Fig. 2). The ROC quantifies the overlap in spike rates obtained when the stimulus is present and when the stimulus is absent. Figure 2A shows example ROC curves, for three units, during visual or whisker stimulation. The dashed line shows what is expected by chance, and the area under the dashed line is 0.5. For the units in the left panel, the area under the ROC curve for whisker stimulation - AUC (Whisker) – is 0.68, indicating that whisker stimulation evoked activity that was significantly (*p* < 0.01; permutation test) higher than spontaneous activity. During visual stimulation the same analysis provides an ROC value of 0.49, indicating no stimulus-driven change in activity. By contrast the unit in the middle panel shows AUC (Visual) of 0.95 and AUC (Whisker) of 0.48, indicating responses to visual modulation only. The unit in the right panel shows AUC (Visual) of 0.73 and AUC (Whisker) of 0.58, indicating excitatory responses to both modalities. In the following, we use the ROC analysis to characterize the sensitivity of spiking activity at 672 contact points of the silicon probe, obtained at 21 recording sites distributed throughout SC.

We expected that visual and whisker responses should be concentrated at different depths within SC, and Fig. 2B shows that this is the case. Each panel shows the distribution of ROC values, during visual or whisker stimulation, as a function of depth below the surface of SC (described in the methods section). Individual sites are color coded according to the following criteria: sites where multiunit activity showed no response to either stimulus (grey symbols), showed response to visual stimulation only (red symbols), whisker stimulation only (blue symbols), or both (black symbols). Figure 2B shows that visually responsive sites are mainly found in superficial layers, whereas whisker responsive sites are in intermediate layers. There is a region of overlap, at depths near 0.6–1.3 mm, where sites could show substantial stimulus driven activity (high ROC values) during both whisker and visual stimulation.

### Comparison of visual and whisker responses at individual sites

**Figure 3:**
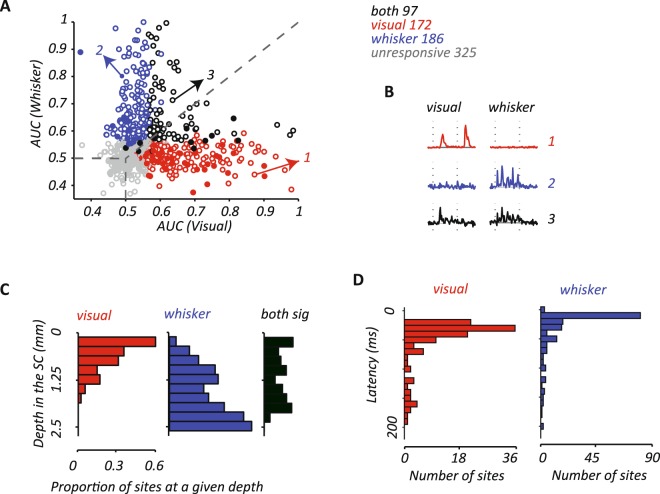
(**A**) Comparison of visual and whisker sensitivity at individual sites for all recorded single- and multiunits. Filled symbols indicate single units recorded using single electrodes (visual n = 39; whisker n = 14; both n = 12; unresponsive n = 43). Unfilled symbols indicate multiunit activity recorded with electrode arrays. Visual-responsive units/sites are indicated by red symbols, and whisker-responsive units/sites by blue symbols. Units/sites responsive to both stimuli are indicated by black symbols. (**B**) PSTHs for the three example sites also shown in Fig. 1. (**C**). Depth distributions of sensory preference for 672 electrode contacts at 21 recording sites. Abscissa shows the depth of the recording site from the surface of SC. Ordinate shows the proportion of all the recording sites recorded at that depth. Visually responsive electrodes (red bars on the left; n = 133) were mainly in superficial layers. Whisker responsive electrodes (blue bars in the middle; n = 172) were mainly in intermediate layers. Significant response to both visual and whisker stimulation was observed across layers of SC (black bars on the right; n = 85). (**D**). Histograms of response latency for visual (n = 118) and whisker stimulation (n = 176). Stimulus-evoked response latency was defined as the first of two consecutive significant PSTH bins compared to the baseline. The median visual latency (34 ms) was longer than whisker latency (15 ms).

The analyses above show that the distributions of sites responsive to visual or whisker stimulation show a limited overlap, but do not tell us the relative strength of responses at each site. Figure 3A compares ROC measurements for visual and whisker responses at individual sites. Here we include, in addition to the multiunit measurements described above, additional single-unit measurements, made using single high-impedance electrodes, from 65 neurons (visual n = 39; whisker n = 14; both n = 12; Fig. 3A; filled symbols). Unfilled symbols indicate multiunit activity recorded with electrode arrays (visual n = 133; whisker n = 172; both n = 85). An area under ROC of 0.5 indicates neural activity is not responsive to the sensory stimulus. Sites that do not respond to either visual or whisker stimulation therefore lie at the lower left of the figure, where both ROC values are around 0.5 (grey symbols). Sites that respond to visual (*p* < 0.05), but not whisker stimulation lie in the bottom right (red symbols). By contrast, sites that respond to whisker (*p* < 0.05), but not visual stimulation lie in the top left (blue symbols). Sites that respond to both visual and whisker stimulation (black symbols) are also present, but these sites generally show strong preference for one of the modalities (few symbols lie in the top right of the plot). Single- and multiunit activity showed similar area under the ROC (AUC) for both whisker and visual responses (whisker AUC 0.62 for single-units vs. 0.68 for multiunit activity; visual 0.66 vs. 0.68). Figure 3B shows example PSTHs for a predominantly visual site, a predominantly whisker site, and a site that responded to both; these sites are also indicated in Fig. 1.

We used the comparison in Fig. 3A to classify units as visual, whisker or both. Figure 3C shows the depth distribution of sites using this classification. To factor out differences in encounter rates, at each depth we calculated the proportion of sites that could be activated by one or both stimuli. Figure 3C reinforces the impression that there is overall segregation of visual and whisker responses in SC. In summary, spiking activity in SC is elevated by whisker or visual stimuli, but rarely both, when those stimuli are presented in isolation. Sites that prefer visual or whisker stimulation show distinct depth distributions, but there is some overlap of these distributions, particularly at depths between about 0.6 and 1.3 mm.

### Temporal overlap between visual and whisker representations

The analyses above show limited spatial overlap between visual and whisker representations in rat SC. Interactions between responses to different modalities will also depend on the temporal overlap, or ordering, of those responses. To characterize the temporal overlap, we calculated response latency to each stimulus when presented in isolation. From PSTHs (here a bin-width of 5 ms) generated for each stimulus, response latency was defined as the first post-onset occurrence of consecutive bins with significant activity (*p* < 0.05). Figure 3D shows the response latency distribution for the visual (left) and whisker (right) stimulations. The latency distribution of visual and whisker stimulation is distinct: the median visual response latency was 34 ms, and the median whisker response latency was 15 ms. For the sites that responded significantly to both visual and whisker stimulation, the median visual latency was 34 ms (n = 32) and the median whisker response latency was 30 ms (n = 46). These response latencies are consistent with previous literature for SC^44,47,61^.

### Suppression of activity during multisensory stimulation

So far we have considered responses to visual and whisker stimuli presented in isolation, and find little evidence for presence of multisensory responses in spiking activity. Stimuli presented to one modality may nevertheless modulate the responsivity of neurons to stimuli presented to the other modality, and we now assess whether and how joint presentation of whisker and visual stimuli affects spiking responses. To increase opportunity for multisensory interactions the onset of visual stimulus was set at 80 ms before the onset of whisker stimulus. They disappeared at the same time. In 11 sessions of recording, a total of 105 recording sites exhibited significant responses to at least one modality. The following analyses include responses from these 105 sites.

**Figure 4:**
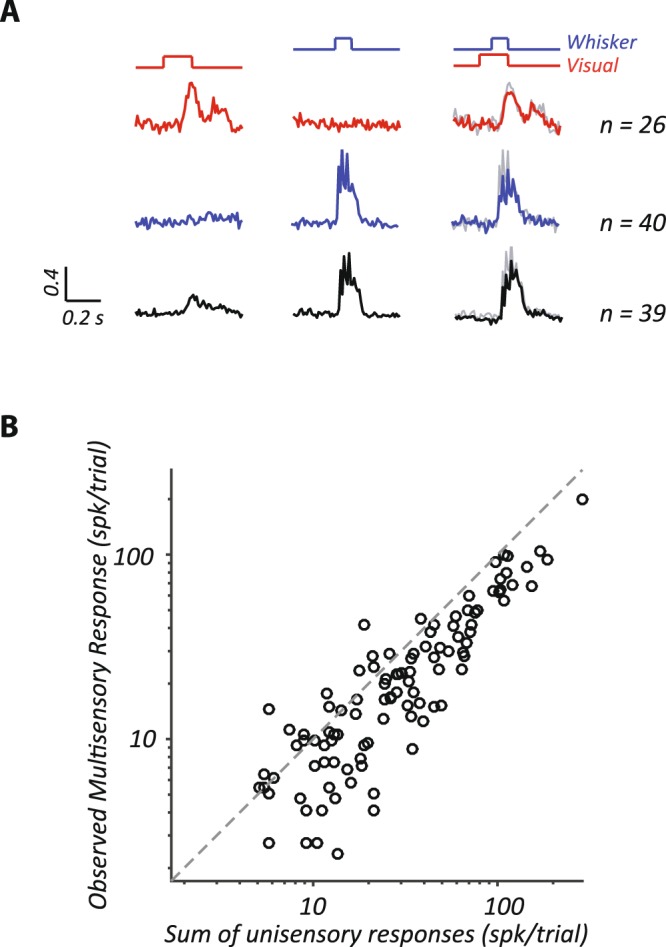
Multisensory stimulation suppresses spiking responses. (**A**) Average PSTHs for visually-sensitive sites (top row), whisker-sensitive sites (middle row) and sites responsive to both visual and whisker stimulation (bottom row). Each column shows responses to maximal visual (left), whisker (middle) and multisensory (right) stimuli. Time course of stimulus presentation is indicated above the relevant PSTHs. Grey lines in the multisensory responses show the response predicted by summation of responses to visual and whisker stimuli when presented alone. Maintained activity was subtracted, and response then normalized to the maximum across stimulus conditions, before averaging. (**B**) Comparison of response to multisensory condition, with that predicted by summation of responses to visual- and whisker stimuli presented alone, for all sites (n = 105). Background activity was subtracted from stimulus-driven activity of all the conditions. Responses generally lie below the unity line (dashed line) indicating suppression of response by multisensory stimulation.

Figure 4 compares the responses of the multisensory and the unisensory stimulation. Figure 4A shows average of normalized PSTHs obtained at maximum visual contrast (left), maximum whisker vibration (middle), and the multisensory combination of both (right). We plot separately the predominantly visual sites (top row), predominantly whisker sites (middle row) and sites that responded to both (bottom row). In each case, the predicted multisensory response (ie. the sum of the unisensory conditions; grey line) is larger than the observed multisensory response, suggesting that there is an overall sub-linear (ie. suppressive) effect of multisensory stimulation. Figure 4B quantifies this effect at individual recording sites, and shows that the observed response is usually less than that predicted by the sum of the two modalities, such that most points lie below the line of unity.

**Figure 5:**
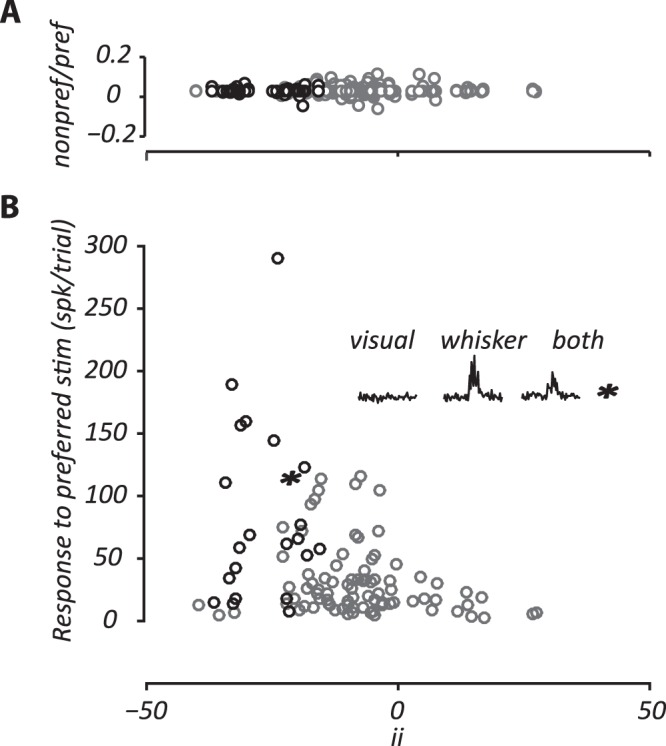
Suppression by stimuli that do not evoke spiking activity. (**A**) Comparison of multisensory “interactive index” (ii) and the relative response to non-preferred stimulus modality (n = 105). The abscissa shows the ratio of response to the non-preferred stimulus to that for the preferred stimulus. (**B**). Comparison of multisensory “interactive index” (ii) and the response to preferred stimulus modality. Inset shows the response of a representative site (star) showing response suppression by multisensory stimulation. Black symbols in A and B show statistically significant “ii” values (Wilcoxon rank sum test performed on responses to preferred and non-preferred stimulus modalities after subtracting spontaneous activity).

A common metric for assessing multisensory integration is the interactive index (“*ii*”; Equation 1), which relates the multisensory response to response in the preferred stimulus modality (the larger of the two unisensory responses). Figure 5 shows the distribution of the interactive index across the measured population for the highest stimulus intensities. For clarity, individual points are not categorized by the preferred sensory stimulus. Figure 5 shows negative interactive indices for the majority of cells, that is, suppression of response during multisensory stimulation. This was particularly the case for whisker-preferring sites and as a population these sites showed a significant suppression of response (*p* < 0.01; nonparametric Wilcoxon rank test). Average interaction index for whisker preferring sites was −11% (n = 40), for visual preferring sites was −6.1% (n = 26) and for sites responsive to both stimuli was −15.7% (n = 39). Analysis of individual sites showed significant interaction indices at 21/105 sites (*p* < 0.05; nonparametric Wilcoxon rank test; black symbols in Fig. 5). All of these showed negative interaction indices; 6 sites were whisker preferring, 2 were visual preferring, and 13 responded to both stimuli. Note that all 13 sites that responded to both stimuli show strong preference for whisker stimulation (mean whisker ROC = 0.84; mean visual ROC = 0.53).

We considered the possibility that facilitative interactions are found in neurons with weak responses to the preferred stimulus, and suppressive interactions are found in those with strong responses, or vice versa. Figure 5B compares interaction index with response amplitude for the preferred stimulus: there is a small yet significant negative relationship (correlation coefficient = −0.24, *p* < 0.01). That is, at sites with weak responses to the preferred modality, there is less suppression from the other modality, and potentially facilitation. We also considered the possibility that interaction indices were found in neurons that show relatively strong responses to the non-preferred stimulus in isolation. Figure 5A shows that the relative response to the non-preferred stimulus, which is always low, does not predict the interaction index. We also calculated the interaction indices for lower intensity stimuli (data not shown). The average interaction index at one-half of the maximum stimulus intensity was smaller, but showed a significant suppression of response (−6.3%, *p* < 0.01; nonparametric Wilcoxon rank test). The average interaction index at one-quarter of the maximum intensity was not significantly different from zero (−2.6%, *p* > 0.05).

In separate measurements, we presented the visual and whisker stimuli synchronously in the multisensory condition and observed similar suppression of responses as that found during the asynchronous multisensory presentation above (data not shown). As for the asynchronous presentation described above, stronger interactions were observed at whisker-preferring sites and as a population these sites showed a significant suppression of response (average −14.9%, n = 43, *p* < 0.01; nonparametric Wilcoxon rank test). Visual preferring sites showed less suppression (−6.3%, n = 23, *p* > 0.05) and sites responsive to both stimuli showed moderate suppression (−8.6%, n = 66, *p* < 0.01). Average interaction index for whisker preferring single units was −1.4% (n = 10), and for neurons responsive to both stimuli was 8.1% (n = 5), neither of which was significant. Average interaction index among visual preferring single units was 6.6% (n = 26, *p* < 0.05; nonparametric Wilcoxon rank test).

In summary, we find that both single-units and multiunit sites in SC are rarely responsive to both visual and whisker stimulation, implying that multisensory units are rare. We also show, however, that there are robust interactions between sensory modalities, even when under unisensory conditions the non-preferred stimulus elicits weak or no spiking response. These interactions are primarily suppressive, and are most prominent at whisker-preferring sites.

### Response surfaces for multisensory stimulation

Our analyses have so far mainly characterized neuronal response to the most intense visual or whisker stimulus, alone or in combination. These responses were drawn from a larger set of responses, to a matrix of stimuli that included all combinations of visual stimulation at each of 5 contrast levels and whisker stimulation at each of 5 vibration amplitudes. In the following, we characterize response across the joint surface. To do this we compared response to the predictions of a simple model of sensory combination (Equation 2). We included for analysis 88/105 sites where the model provided good predictions (*r*^2^ > 0.6). These included 15/26 (58%) visually responsive sites, 30/40 (75%) whisker responsive sites and 29/39 (74%) sites that responded to both stimuli. Most of the excluded sites responded only to stimuli of the highest intensity, and even that response was small (mean whisker ROC = 0.587; mean visual ROC = 0.594).

**Figure 6:**
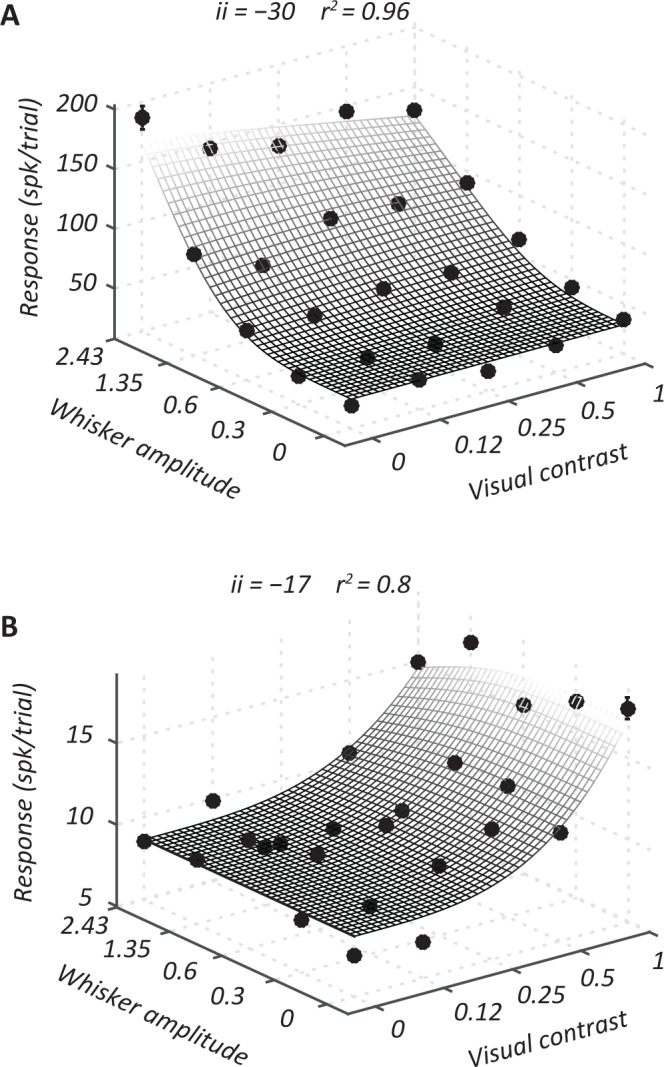
Model based characterization of responses to combinations of visual and whisker stimulation. (**A**) Responses of a representative whisker-preferring site to a matrix of visual-contrast and whisker stimulation amplitude. Spontaneous activity has been subtracted. For clarity, only mean responses are plotted; indicative s.e.m. is shown for one stimulus condition in each case. (**B**). Same as A, but a visual preferring site. Surfaces show the predictions of the generalized normalization model (see Methods).

Figure 6 shows response of a representative whisker-preferring site (Fig. 6A) and a visual-preferring site (Fig. 6B) to the set of 25 possible stimuli. Suppression from the non-preferred stimulus modality is observable at high intensities (the curves bend down in the far corner of the graph). The interpolated surface shows the best predictions of the model. The model proposes that there are two types of input to each neuron. The numerator of the model is the sum of excitatory inputs from whisker and visual sensory pathways. The denominator provides a parallel suppressive input, which is also driven by whisker and visual inputs. The relative strength of excitatory visual and whisker inputs (numerator) is captured by the parameter *w*_1_, with higher values indicating stronger visual input. Similarly, the relative strength of visual and whisker inputs to the suppressive, or ‘normalization’ pool (denominator) is captured by *w*_2_.

**Figure 7:**
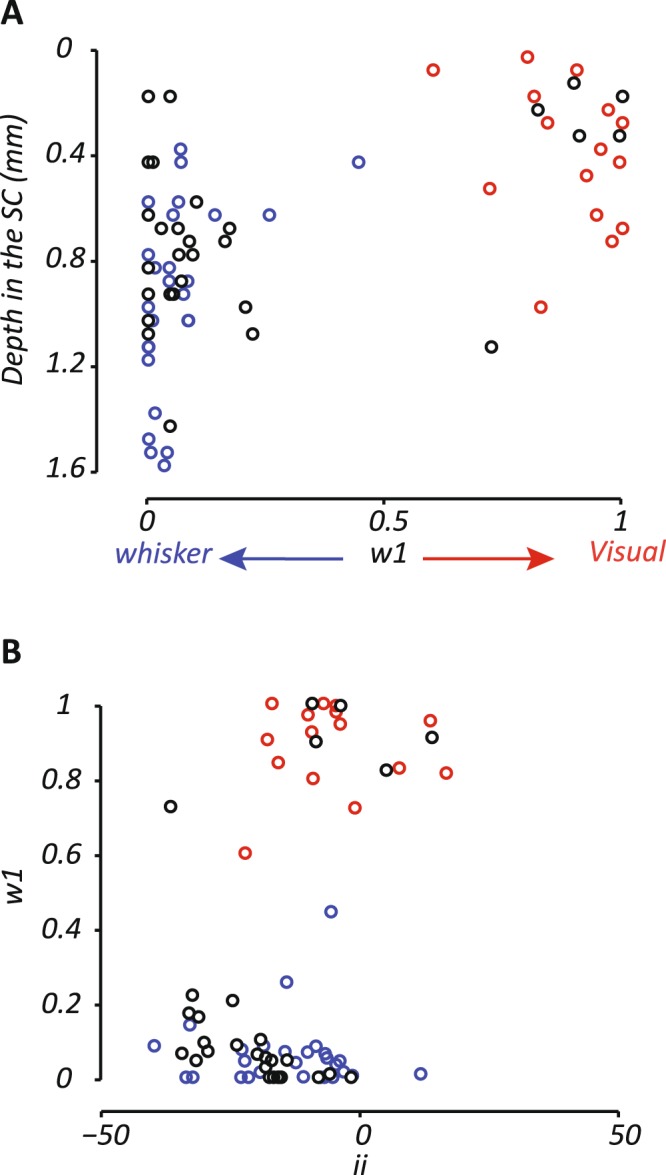
Suppressive multisensory interactions are strongest at whisker preferring sites. (**A**) Depth dependence of model-derived weights for visual- and whisker input (*w*_1_). *w*_1_ of 1 indicates excitatory drive only from visual input; *w*_1_ of 0 indicates only whisker input. Sites with poor model fits (*r*^2^ < 0.6) were excluded from the analysis. The depth dependence of excitatory visual and whisker inputs is similar to that obtained from AUC analyses. Symbol color is defined by the category (visual - red, whisker - blue, both - black) obtained from the AUC analyses in Fig. 2. (**B**) Comparison of *w*_1_ values and the “interactive index” (ii). Substantial negative interactive indices are primarily at sites with strong excitatory responses to whisker stimuli (ie. *w*_1_ near 0).

Figure 7A shows the distribution of *w*_1_. To allow comparison with the ROC analyses above, the symbol color shows the category (visual, whisker, both) defined by those analyses. Happily, sites with *w*_1_ less than 0.5 (whisker) were also defined as whisker preferring sites in the ROC. Sites with lower *w*_1_ (whisker) are generally located deeper in the SC than sites with higher *w*_1_ (visual). Also consistent with the above analyses, Fig. 7B shows negative interactive indices are more pronounced among sites that prefer whisker stimulation (mean “ii”: −14.4, n = 59). Visual sites (*w*_1_ > 0.5) show smaller interactive indices (µ −7.8, n = 29).

**Figure 8:**
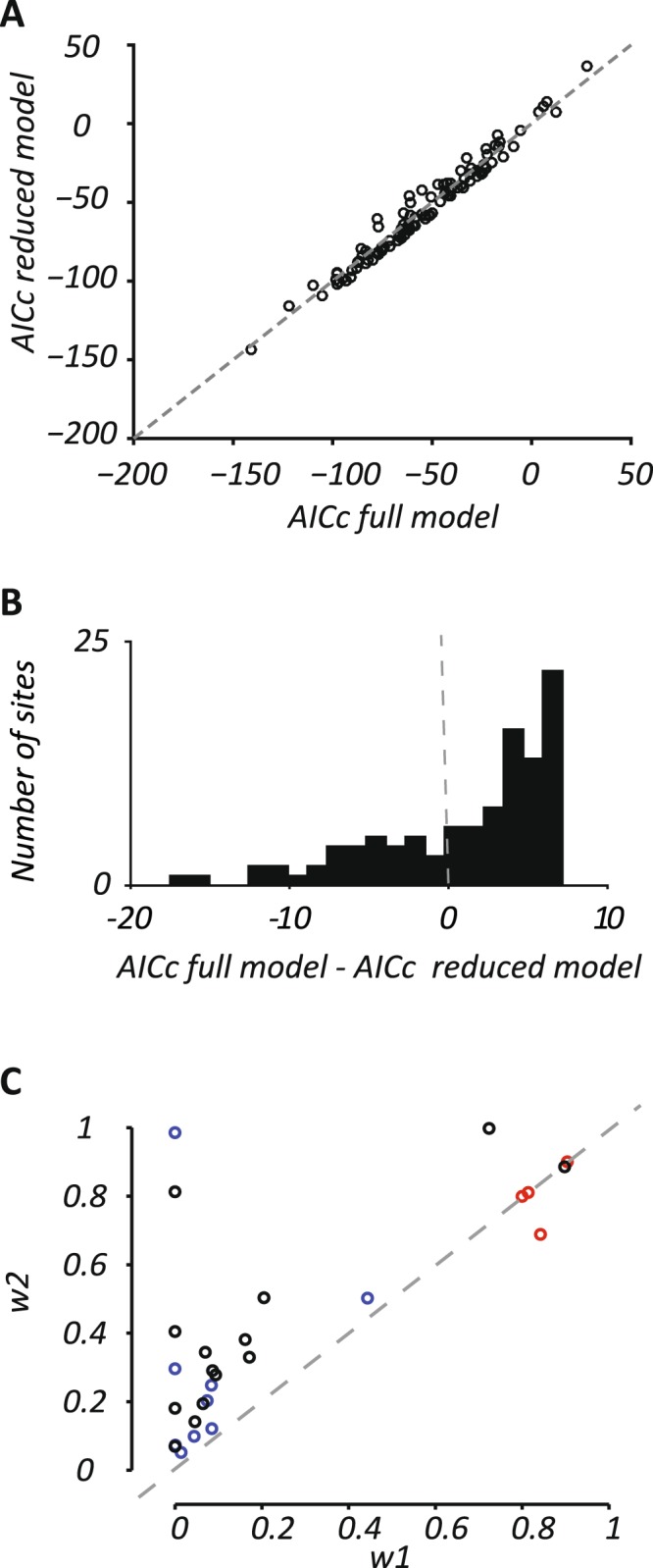
Multisensory contribution to suppression. (**A**,**B**) Comparison of model performance for models with (‘full model’) and without (‘reduced model’) a suppressive component (n = 105). The best model is that with the lowest AICc values. (**A**). Each point compares the AICc value for the two models: data points above the unity line (dashed line) indicate the full model provided better predictions having accounted for the increased number of parameters. (**B**). Histogram of the difference between AICc values for the full and reduced models. Negative values indicate that the full model provided better predictions than the reduced model. (**C**). Comparison of weights applied to each sensory modality for the excitatory drive (*w*_1_) and suppression (*w*_2_). Sites included only where the model provided good predictions (r^2^ > 0.6), and AICc analyses showed better fits for the full model. The suppressive weight (*w*_2_) like *w*_1_, ranges from 0 to 1, with a value of 1 indicating that suppression was only sensitive to visual stimuli.

The model allows us to estimate the relative weight of visual and whisker inputs to suppressive mechanism (*w*_2_), but this will only be meaningful where the model is well constrained. To restrict analyses to informative sites, we calculated the corrected Akaike Information Criterion (AICc; see Methods) for the full model, and a reduced model in which the denominator was removed. The best model is that with the lowest AICc values. Figure 8A compares the AICc values for the two models at individual sites (for the 105 sites that showed significant responses to one of the modalities). Many points lie below the line of unity, indicating lower AICc scores for the reduced model. For analysis of suppressive mechanisms (*w*_2_, in the denominator), we therefore included only those sites that show added benefit of these terms (that is, AICc was lower for the full model than the reduced model; Fig. 8B). Figure 8C compares, for those sites where the full model offered better predictions, the distributions of *w*_1_ and *w*_2_. Most points are clustered to the left of the plot, indicating that sites with predominantly whisker input were more likely to show suppressive interactions. Further, most points lie above the line of unity, indicating relatively stronger contribution of visual input to the suppressive mechanism (denominator) than the excitatory mechanism (numerator). The same analyses were also performed for all sites regardless of whether the full model offered better predictions. Again, sites with predominant whisker input were more likely to show suppressive interactions.

## Discussion

We established the dorsoventral distribution and interaction of visual and whisker responses in superior colliculus of rat. Spiking activity was elevated by whisker or visual stimuli, when those stimuli were presented in isolation, but rarely both. Visual and whisker responses were differentially distributed across the depth of SC, with visually responsive sites primarily in superficial layers and whisker responsive sites in intermediate layers. Measurements of response latency revealed distinct temporal dynamics for visual and whisker representations, such that visually driven responses lagged whisker-driven responses, at least under these measurement conditions. Despite the general lack of overt responses to both modalities at single recording sites, we found robust and primarily suppressive cross-modal interactions.

Simultaneous recordings with multichannel linear probes allowed large-scale characterization of visual and whisker responses. We found limited overlap of visual and whisker representations within SC. From about 0.6 to 1.3 mm below the surface there was a mixture of both single- and multi-unit activity that responded robustly to either visual or whisker stimuli, but not both. The spatial overlap, without overt multimodal responses, is consistent with “areal convergence” of sensory signals in the SC^1^. On individual shanks in single penetrations (e.g. Fig. 1D) we often saw interdigitation of sites that were responsive to each modality. Consistent with the overall pattern, these interdigitated sites were located in the intermediate layers. The location of responsive whisker and visual sites in our simultaneous recordings is consistent with previous anatomical and functional studies of whisker and visual representations in SC^36,43,45,47,62–64^.

We used large visual stimuli and multi-whisker vibrations to maximize the chance of observing responses from either modality by activating as many units on the multichannel probes as possible. Nevertheless, we cannot rule out the possibility that stronger multi-modal responses might be observed if the visual stimuli were optimized for individual neurons. Our large stimuli were capable of producing spiking responses, as has also been reported elsewhere^6,15,43–45,47^. Neurons in SC have large whisker receptive fields and respond more to simultaneous multi-whisker stimuli than to single whiskers^44,65^. While the visual sensitivity of neurons in the uppermost layers of SC is likely to be decreased using full-field visual flashes^42^, receptive fields in lower layers may be as large as our stimuli^47,66^. Indeed neurons in the deep layers of mouse and cat SC have cells with large receptive fields approaching the size of our visual stimuli^47,66,67^. In addition, Bytautiene *et al*.^68^ showed that at least neurons in the superficial layers of rat SC are capable of integrating stimuli over a larger area than that implied by the classical receptive field – at least in anesthetized rats, these upper layers of SC are the main candidate for excitatory inputs to deep layers of SC^69^. Thus while our stimuli may be much larger than optimal for neurons in the superficial layers of SC, they are likely to be closer to optimal for deeper neurons. Finally, we note that to obtain large numbers of trials we presented brief stimuli in rapid succession. It is well known that neurons in SC are susceptible to habituation, and measurements of multisensory interaction may depend on habituation state^43,70,71^. Previous work on visual habituation in anesthetized rat SC shows that habituation can be profound for white spots against a dark background, but much of that is light adaptation in the retina^71^; we presented stimuli against a mean grey background and light adaptation is not likely to be pronounced in these conditions. Hemelt and Keller (2007) showed little adaptation of whisker responses in anaesthetised rat SC for stimulation frequencies below about 5–8 Hz. Indeed, different sensory modalities may show different habituation dynamics, and this may be a useful target for future work.

The SC is involved in both approach and avoidance behaviors. Approach behavior seems to depend on crossed descending tecto‐reticulo‐spinal projections from the lateral SC^72^, while a medial, uncrossed pathway is involved in avoidance and flight behavior^73^. Consistently, the medial and lateral parts of SC show substantial differences in the pattern of inputs from other brain areas^74^ that are in accord with their differential contribution to approach and avoidance behaviors. The medial part of SC represents the upper visual field (a likely source of aerial predators) and whisker responses are largely absent from medial SC. The lateral SC represents the lower visual field (a likely source of prey or mates) and whisker responses are stronger in lateral SC^41,73–76^. Our recordings were made from lateral SC (6.8 mm posterior to bregma, 1.5 mm lateral to the mideline) to maximize the chance of recordings from units with central to lower visual receptive fields and on the macro vibrissae. A useful target of future work would therefore be to establish whether interactions between whisker and visual responses differ between the medial and lateral SC, or whether interactions between medial and lateral SC may depend on sensory modality. We note that whiskers are not the only somatosensory inputs to SC – for example there are nociceptive neurons in rat SC, which are not responsive to whisker stimulation^77^ – and it would be interesting to know whether cross-modal interactions depend on the type of sensory signals that the neurons are representing.

We characterized spiking activity in response to combined visual and whisker stimulation at multiple stimulus intensities and found that each modality generally suppressed the responses obtained through the other modality. Suppressive interactions between sensory modalities have previously been observed in some neurons in SC^14–17,26^ and other areas of the brain^78–80^. In SC of cat, those neurons that show suppressive impact of multisensory stimulation are often only influenced by stimuli from one modality during unisensory presentations^14^. This observation in cat is consistent with our observations in rat: the response to the non-preferred stimulus was always low, and the suppressive influence of a seemingly ineffective stimulus only became evident during multisensory presentation. These findings have an important implication: given that the suppressive influences of one modality onto the other are evident only when the stimuli are combined, it is likely that the proportion of multisensory neurons is generally underestimated. When stimuli from different modalities are presented sequentially, they are ineffective in identifying suppressive interactions except in those neurons that have high and regular spontaneous activity^1^.

Our results, obtained from isoflurane-anesthetized rats, show that stimulation of one sensory modality generally suppressed the responses obtained through the other modality. It is known, however, that anesthesia alters neuronal excitability in multiple brain areas, and it remains an open question whether and to what extent these results would generalize to awake animals. For example, anesthesia modulates the flow of information from cortex to the SC in mice^81^ and visual responses in the deeper layers of SC are inhibited in anesthetized rats, requiring disinhibition (suppression of GABAergic inhibition) for their visual response to be revealed^69,82^. Our measurements may therefore underestimate the level of interaction between modalities.

The multimodal suppressive interactions indicate that “unimodal” neurons derive suppressive input from either multisensory neurons, or pools of neurons individually sensitive to each modality. If this suppression arises in the SC, it may reflect pooling of inputs from neurons within SC, but it may also reflect inputs from other areas, particularly those cortical areas that show more overt convergence of multimodal signals. Multisensory integration in SC of cat is partially mediated by cortical areas (for example Jiang *et al*.^83^; Alvarado *et al*.^84^). Similar areas are likely to be present in rat neocortex^6,46,85–87^. Indeed, spiking activity in parietal cortex of rat shows sub-linear or suppressive interactions between visual and whisker modalities^6^, similar to what we observe in SC. Similarly, a visuo-tactile area between the rostral primary visual cortex and the caudal primary somatosensory cortex of the mouse shows sublinear integration of post synaptic potentials^88^. Other work in striatum also found that multisensory visual and whisker stimuli were integrated sublinearly^89^.

One reason for the primarily suppressive or sublinear cross-modal interactions in our study and others may be that these signals are rarely paired in experience. While rats mainly use whisker cues for navigating and foraging in the burrow, vision appears more important for representation of distal stimuli (eg. detection of avian predators) in the open field. Our results are compatible with the key predictions of recent modelling by Cuppini *et al*. (2018), who show that cross-modal stimuli should facilitate units in SC only when the stimuli have been paired during experience^90–93^. In the absence of paired experience, the circuit might extend its internal logic to process any two cues as competitive^94^, and suppress unisensory responses to the preferred stimulus. Cuppini *et al*. (2018) suggest that at least some of the competitive interactions in SC are due to the processes that take place within cortex which are then relayed to the SC. Cortical input to SC is likely to be particularly important in making sensory processing in SC dependent on attentional state and previous experience^81,95–97^ and the suppressive multisensory interaction that we observe may be part of a mechanism for cortical selection of sensory processing in SC, helping activity evoked through one modality to suppress noise in the other modality^98^.

The normalization framework we and others have used is analogous to Bayesian accounts of multisensory integration, where optimal multisensory integration takes the reliability of each source into account: greater weight is given to more reliable sensory cues^99–101^. SC is important for orienting behavior towards or away from external events^1,2,102,103^, including control of head^104^, eye^105^ and whisker movements^65^. Whisker responsive cells in SC are thought particularly important in mediating approach behaviors^62,73,102,106^. Avoidance or defensive responses evoked by threatening stimuli are also associated with SC. Microstimulation of SC can cause defensive reactions including freezing and escape^107,108^. Understanding the mechanisms that support cross-modal integration in SC neurons can provide insights into the role of SC circuits in these ecological functions.
